# Using evolutionary data to make sense of macromolecules with a “face‐lifted” ConSurf

**DOI:** 10.1002/pro.4582

**Published:** 2023-02-21

**Authors:** Barak Yariv, Elon Yariv, Amit Kessel, Gal Masrati, Adi Ben Chorin, Eric Martz, Itay Mayrose, Tal Pupko, Nir Ben‐Tal

**Affiliations:** ^1^ George S. Wise Faculty of Life Sciences, Department of Biochemistry and Molecular Biology Tel Aviv University Tel Aviv Israel; ^2^ Department of Microbiology University of Massachusetts Amherst Massachusetts USA; ^3^ George S. Wise Faculty of Life Sciences, School of Plant Sciences and Food Security Tel Aviv University Tel Aviv Israel; ^4^ George S. Wise Faculty of Life Sciences, The Shmunis School of Biomedicine and Cancer Research Tel Aviv University Tel Aviv Israel

**Keywords:** ConSurf, evolutionary conservation, function prediction, functional regions

## Abstract

The ConSurf web‐sever for the analysis of proteins, RNA, and DNA provides a quick and accurate estimate of the per‐site evolutionary rate among homologues. The analysis reveals functionally important regions, such as catalytic and ligand‐binding sites, which often evolve slowly. Since the last report in 2016, ConSurf has been improved in multiple ways. It now has a user‐friendly interface that makes it easier to perform the analysis and to visualize the results. Evolutionary rates are calculated based on a set of homologous sequences, collected using hidden Markov model‐based search tools, recently embedded in the pipeline. Using these, and following the removal of redundancy, ConSurf assembles a representative set of effective homologues for protein and nucleic acid queries to enable informative analysis of the evolutionary patterns. The analysis is particularly insightful when the evolutionary rates are mapped on the macromolecule structure. In this respect, the availability of AlphaFold model structures of essentially all UniProt proteins makes ConSurf particularly relevant to the research community. The UniProt ID of a query protein with an available AlphaFold model can now be used to start a calculation. Another important improvement is the Python re‐implementation of the entire computational pipeline, making it easier to maintain. This Python pipeline is now available for download as a standalone version. We demonstrate some of ConSurf's key capabilities by the analysis of caveolin‐1, the main protein of membrane invaginations called caveolae.

## 1 INTRODUCTION

The evolutionary rate per site in protein, DNA, and RNA sequences reflects a balance between opposing effects. There is the overall tendency to change, which is driven by mutations. Sites evolving under mutation pressure alone are referred to as evolving neutrally. Some sites experience positive selection, driving them to rapidly evolve and to generate, for example, novel recognition sites or to avoid recognition by drugs or the host immune system. In contrast, some sites are subject to a purifying selective regime to ensure that structure and/or function are retained and thus evolve slowly resulting in evolutionarily conserved regions. Thus, mapping the evolutionary rates per sites onto the sequence or the structure of a macromolecule can reveal functionally important regions that are relevant targets for follow‐up research.

Exploiting evolutionary data to detect functional regions in proteins and in nucleic acids is very commonly used (Capra et al. 2009; del Sol Mesa et al. 2003; Gallet et al. 2000; Landgraf et al. 2001; Lichtarge et al. 1996a; Lichtarge et al. 1996b; Lichtarge et al. 1997; Valdar 2002). Evolutionary rates are often used in genomics analyses to predict the pathogenicity of single‐nucleotide variants identified in patient samples (Labes et al. 2022 and references therein). They can also be used in protein engineering efforts (Pavelka et al. 2009). Methods for estimating evolutionary conservation that are based on consensus and relative entropy approaches (Sander & Schneider 1991) can be misleading when, for example, there is a bias toward a specific taxonomic group. In addition, such approaches do not take into consideration the similarity between the amino acids (or nucleotides). ConSurf (Armon et al. 2001) and related methodologies (Huang & Golding 2014; Huang & Golding 2015; Morgan et al. 2006) are superior to such methods as they account for the evolutionary relationships between homologues represented as a phylogenetic tree.

ConSurf provides a means to explore the evolutionary conservation pattern of proteins and nucleic acids. Given a query sequence of a protein or nucleic acid, the ConSurf pipeline automatically constructs a multiple sequence alignment (MSA). Using probabilistic evolutionary models, the pipeline then estimates the evolutionary rate per site in the alignment by explicitly taking into account the phylogenetic relationships among the homologues, as reflected in the tree, and the exchangeability probability between any pair of amino acids or nucleotides (Mayrose et al. 2004; Pupko et al. 2002). The statistical robustness of the pipeline facilitates the differentiation between a genuine conservation signal due to purifying selection versus apparent conservation due to insufficient evolutionary signal. As a credibility measure, ConSurf assigns confidence intervals around the estimated rates. ConSurf then clusters the rates into evolutionary grades and maps these grades onto the sequence and/or structure of the query. Mapping of the grades onto the macromolecule's structure is particularly insightful because it often shows surface clusters of evolutionarily conserved sites. These tend to be biologically functional regions that, for example, mediate interactions with ligands, carry out enzymatic catalysis, or mediate oligomerization.

Here, we report on the improvements in ConSurf since the last release in 2016 (Ashkenazy et al. 2016). These include re‐implementation of the pipeline in Python to facilitate maintenance, addition of the capacity to map rates onto an AlphaFold model structure based on its UniProt ID (The UniProt Consortium 2022), embedding of two fast and highly efficient homolog detection methods (HMMER [Finn et al. 2011] and MMsecs2 [Steinegger & Söding 2017]) to keep up with the rapid increase in sequence databases, and the introduction of a new and more intuitive user interface.

## 2 THE PIPELINE

The ConSurf pipeline, shown and described in detail in the OVERVIEW section of the web‐site, offers multiple alternative procedures to analyze proteins and nucleic acids. In the most convenient and commonly‐used alternative, the scholar provides only the sequence or structure of the query protein or nucleic acid. The analysis is based only on the sequences of the query and its homologues, and structural information is used only for visualization. However, when possible, we recommend starting from a query structure (in PDB or mmCIF format) for intuitive visualization of the conservation pattern. For example, mapping the conservation pattern onto a structure makes it easy to differentiate between stability‐conferring and functional residues; the former tend to be buried inside the core of the protein, whereas the latter tend to reside on the surface. In this respect, it is noteworthy that AlphaFold structures are now available for most UniProt proteins (Callaway 2022). We highly recommend using these model structures rather than the corresponding protein sequences.

ConSurf analysis is based on MSA of the query and homologues and a corresponding phylogenetic tree. Advanced scholars may upload their own carefully constructed MSAs in FASTA or other widely‐used formats. The query must be included in the alignment as well as in the tree. It is noteworthy that the computation time scales with MSA size. Thus, we recommend that the MSA be limited to no more than 300 homologues. With more sequences, the Rate4Site algorithm, used to calculate the evolutionary rates per site, shifts to a slow version that may take days to complete.

In the most convenient analysis, given the query sequence or structure, ConSurf automatically searches for homologues, clusters them based on sequence similarity using CD‐HIT (Li & Godzik 2006), and selects (approximately) a predefined number of representatives, 150 by default. These are multiply aligned, and the MSA is used as input to a Rate4Site calculation of the evolutionary rate per site (Pupko et al. 2002). The rates are then binned to nine grades, with one being the fastest (highly variable), five being average, and nine being the slowest (highly conserved). These grades are translated to a color code and mapped onto the query sequence and/or structure for visualization. The homolog selection depends on the choice of sequence database and search parameters, such as minimal and maximal sequence similarity, and number of search iterations. Defaults are automatically suggested, which often work, but scholars are encouraged to try other possibilities.

## 3 IMPROVEMENTS COMPARED WITH THE 2016 VERSION

### 3.1 New user interface

**FIGURE 1:**
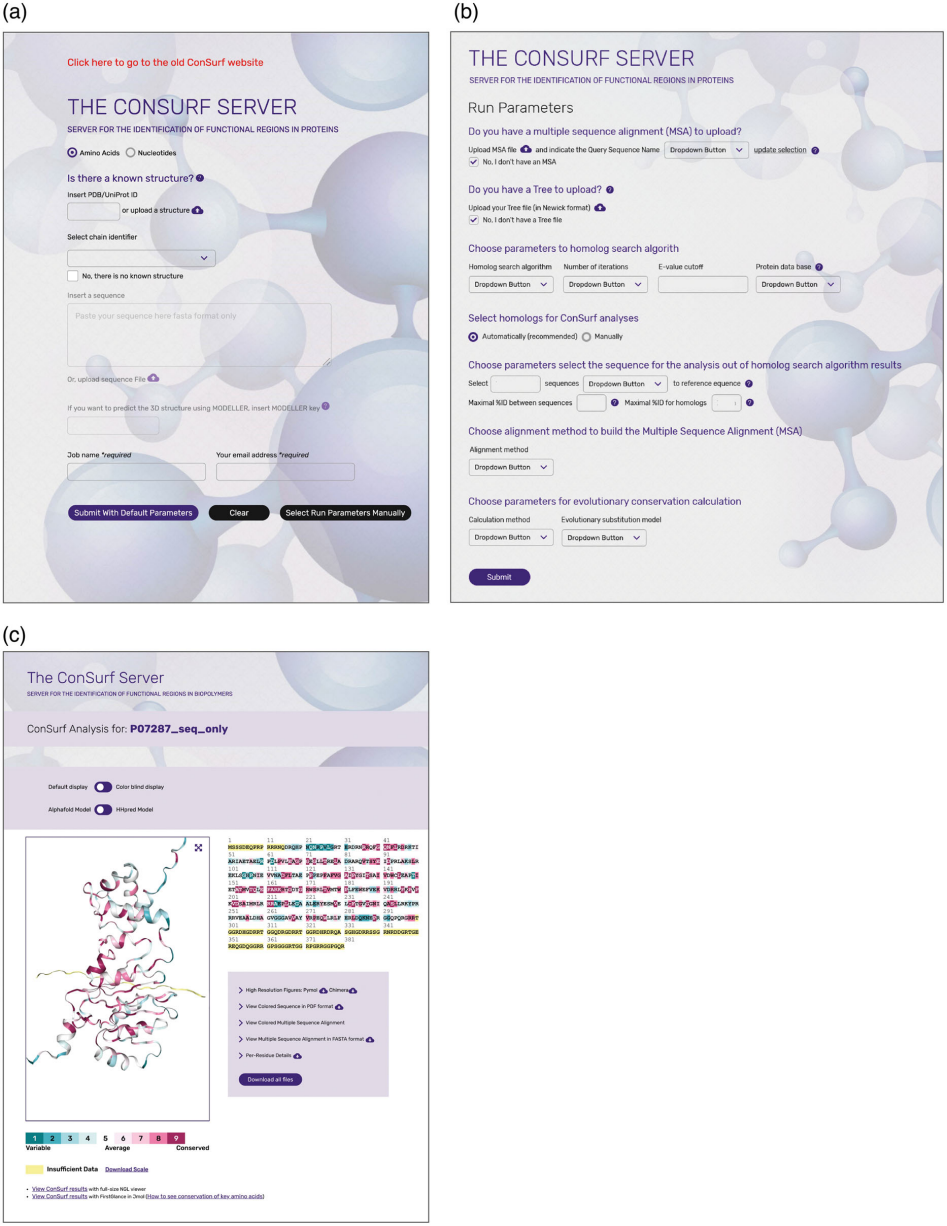
ConSurf calculation with a protein query. (a) The input page. Scholars can provide a query protein structure based on its PDB ID, an AlphaFold model based on its UniProt ID, or by uploading a coordinate file. Calculations can also start by pasting the amino acid sequence of the query protein into the query box. (b) The run parameters page. The scholar can use HMMER (default), MMseqs2, or one of three BLAST‐based homolog search algorithms with UniRef90 and other databases. (c) The results page. The scholar can choose different views of the conservation‐colored structure, alternate between AlphaFold and HHPred models and between standard and color‐blind scales, inspect non‐covalent interactions, map the scores on multiple chains in homo‐oligomers, download pre‐made session files of both PyMOL and ChimeraX, and prepare animated gifs.

A new and much improved user interface has been implemented that makes it easier to start a calculation and to visualize the results (Figure 1). The interface allows the scholar to start a calculation using the PDB ID of a query protein of known structure or the UniProt ID of a protein with an AlphaFold model structure. Default parameters are automatically included to facilitate rapid preliminary analysis, but scholars can choose other parameters depending on the query and question at hand. Brief explanations of the parameters are available by clicking on the question marks embedded in the interface, and more details are provided in the “OVERVIEW” and “FAQ” tabs of the website. When a calculation is started for a protein that is already included in the ConSurf‐DB repository (Ben Chorin et al. 2020), a message pops up to notify the scholar about this availability. The new interface also allows users to cancel an unnecessary execution, reducing workload on the cluster. Of note, the main results page shows the query structure (when available) and sequence with the projected conservation pattern.

### 3.2 Ability to start a query with an AlphaFold model

ConSurf is now configured to allow a calculation to be started using an AlphaFold model. For queries from the AlphaFold database (Tunyasuvunakool et al. 2021) the scholar can provide the UniProt ID of the query in the box below “Is there a known structure” (Figure 1a).

### 3.3 Homolog search

A ConSurf calculation critically depends on having a large and diverse set of homologues that sufficiently cover the sequence space. The 2016 pipeline offered the BLAST‐based search methods PSI‐BLAST(Altschul et al. 1997; default) and CSI‐BLAST (Angermüller et al. 2012), which were sufficient at the time. However, the enormous growth of sequence databases requires the use of more advanced search tools such as those based on hidden Markov models. Thus, the ConSurf pipeline now embeds both HMMER and MMseqs2 searches for protein queries, making the former the default (Figure 1b). By default, the search for homologues is conducted against the UniRef90 database, which contains UniProt representatives filtered to 90% sequence identity; however, other sequence databases are also offered. We also added the HMMER search for nucleic acid queries, setting it to be the default.

### 3.4 Nucleic acids analysis

In principle, the previous version of ConSurf was capable of handling nucleic acids. However, nucleic acid sequence analyses were impractical because the previous pipeline failed to construct a large enough and sufficiently diverse set of homologues for RNA and DNA. Unfortunately, with the continuous increase in database sizes, the nhmmer (Wheeler & Eddy 2013) search for homologues became too demanding, and the previous pipeline was unable to handle nucleic acid queries. The architecture of the new pipeline is suitable for such memory‐ and time‐consuming processes, and can therefore successfully analyze nucleic acids. In this respect, it is noteworthy that to analyze RNA, we recently suggested protocols for building suitable MSAs using external resources (Rubin & Ben‐Tal 2021)

### 3.5 Visualization

ConSurf is most commonly used to analyze protein structures. When the structure of the query protein is known, or can be modeled, the main result page includes the conservation grades mapped onto the structure, using an improved color bar (Figure 1c). In this color bar, recently used in the ConSurf database (ConSurf‐DB; Ben Chorin et al. 2020), the difference between conservation grades is better distinguished. The conservation‐colored structure can be visualized in the fast NGL viewer, which allows zooming in on the interactions of the query with ligands, highlighting hydrogen bonds, etc.

ConSurf also offers visualization via FirstGlance in Jmol, which has had numerous enhancements since 2016. FirstGlance offers a “Contacts and Non‐Covalent Interactions” tool: a scholar can select (by clicking on, or finding by name) any moiety, and the interacting residues are isolated and colored by conservation. The view can be simplified by restricting to hydrogen bonds, apolar interactions, etc. Illustrated step by step instructions are provided in: https://proteopedia.org/w/FirstGlance/Visualizing_Conservation. In this visualization, the user can click on an amino acid to reveal its conservation grade, regardless of the color scheme applied. When the structure data (PDB file) specifies a quaternary assembly, it is automatically constructed by FirstGlance, colored by conservation. An example is the binding of p53 to DNA (PDB ID 5mct). The coordinates of 5mct have only one strand of a DNA double helix. FirstGlance automatically constructs the complete double helix. This reveals that the amino acid residues S241, A276, and R280 contact DNA through hydrogen bonds and are highly conserved (http://firstglance.jmol.org/fg.htm?mol=5mct_A_consurf_firstglance.pdb). FirstGlance also makes it easy to generate an animation (i.e., a GIF) of the molecular view in just a few mouse clicks. Such animations can be dropped into presentation slides or displayed on websites. In a change since 2016, the animation‐generating mechanism is now server‐based and much more reliable, and an Animation Kit is provided, enabling greater control when desired.

### 3.6 Homo‐oligomeric proteins

In a ConSurf calculation for a PDB entry with multiple identical chains, the conservation grades, calculated for one, arbitrarily chosen chain, are automatically mapped to the rest of the chains. By default, the conservation grades are mapped only onto the selected chain, but both the NGL viewer and FirstGlance in Jmol are now configured to present the grades on any, some, or all of the other identical chains.

### 3.7 Readily available PyMOL and UCSF ChimeraX sessions

In the old version of ConSurf, scholars were given a modified PDB file of their query with the conservation grades in the temperature factor column, and scripts that allowed the query to be colored by conservation grades. Recently, we replaced this cumbersome procedure with an option to download pre‐made PyMOL (DeLano 2002) and UCSF ChimeraX (Pettersen et al. 2021) sessions of the query structure, color‐coded by conservation. To create high‐resolution images, the scholars need only to open the files with PyMOL or UCSF ChimeraX and save them as figures. The scripts and modified PDB files for PyMOL and ChimeraX remain available should the scholar prefer using the older method. We have also added a script for coloring residues according to their conservation grades using MAESTRO (Schrödinger Release 2022–2023: Maestro, Schrödinger, LLC, New York, NY, 2021).

### 3.8 Compatibility with the mmCIF format

Previous versions of ConSurf could only accept PDB files as input structures. Although the older PDB format is accessible and easy to read, the fixed width of the columns limits the number of atoms and chains that can be included within the structure. Structures of large macromolecules, such as the ryanodine receptor, the ribosome, the nuclear pore complex, and virus capsids, are only available in the mmCIF format (Westbrook & Fitzgerald 2003). The mmCIF format is more cumbersome and difficult to read than the PDB format, but it is not constrained by the number of atoms or chains in the structure. The new ConSurf pipeline can read and parse the mmCIF format, allowing conservation analysis of entire sections of the PDB that were previously unavailable.

### 3.9 A new python pipeline

The ConSurf web server was constructed from a patchwork of different Perl scripts by many generations of researchers, and the code in its entirety was never optimized. It had many repetitions, suffered from poor memory usage, and was unstable at times. Additionally, some of the features we implemented in the past had become unavailable following updates and migrations to different machines. We therefore decided that it was no longer sufficient to update the old pipeline, but that it was necessary to completely rewrite the ConSurf pipeline anew using Python.

The new ConSurf pipeline has been consolidated into a single Python script, which sends the more laborious calculations (e.g., homolog search, multiple sequence alignment, and Rate4Site) to our CPU cluster. This not only reduces the load on the head‐node of the CPU cluster but enables failures to be detected in the subprocesses. As a result, some processes that were too demanding for the old pipeline can now be readily handled by the new Python‐based pipeline. For instance, searching for homologues of a DNA or RNA sequence using nhmmer was previously too demanding; many of the searches never ended and eventually overloaded the CPU cluster. With the new pipeline, this failure is quickly remedied and additional memory is allocated for the subprocess.

In PDB format, atoms are designated ATOM or HETATM. The term ATOM is used for all the atoms in the standard residues of protein, DNA, or RNA, and the term HETATM is used for hetero‐atoms in non‐standard residues (and carbohydrates, substrates, ligands, solvent, metal ions, and other groups). The old pipeline took into account only residues that were marked as “ATOM”. The new pipeline also uses residues marked as HETATM, thereby expanding the ConSurf coverage.

The new pipeline also revived secondary ConSurf functionalities that had been lost over time. For example, with the new pipeline it is again possible to conduct ConSurf analysis with homologues extracted from a user‐specified subtree of the phylogeny. To this end, the scholar should click on the “View MSA and phylogenetic tree using WASABI” link (under the “Homologues, Alignment and Phylogeny” menu) to view the phylogenetic tree. The scholar should then mark an internal node at that tree, representing the root of the subtree of interest (this subtree should include a sufficient number of homologues), and open the WASABI menu using a right mouse click. Selection of the option “run ConSurf on subtree” will open another window with a new ConSurf run for the homologues in the subtree. This functionality can be useful to detect functional regions that are unique to homologues in the subtree, (i.e., specific traits shared only by subfamilies).

Structure prediction is another feature that was revived in the new pipeline. When submitting a ConSurf run that is based on a protein sequence rather than a structure, the pipeline automatically searches the AlphaFold database for an available structure. In addition, ConSurf offers structure prediction using the HHpred (Söding et al. 2005; Zimmermann et al. 2018) and MODELER (Sali et al. 1995; Sali & Blundell 1993; Webb & Sali 2016) computational tools. The MODELER key (freely available for academic use) is required if the user wishes to use this tool. As mentioned above, in the absence of experimentally determined structure, we highly recommend using a model structure. Fully automated tools and databases, such as AlphaFold, RoseTTAFold (Baek et al. 2021), and the ESM protein language model (Lin et al. 2022), readily provide protein model structures. For an RNA query sequence, the pipeline provides a ViennaRNA(Lorenz et al. 2011) prediction of the secondary structure.

### 3.10 Standalone version of ConSurf

Along with the web server version, the new pipeline is available as a standalone Python script that can be executed locally on any Unix‐based system. To execute the standalone script, the local system must have: (1) Python 3.8 or newer, (2) the latest Biopython module installed inside the Python environment including all of its dependencies, (3) at least one of the applications used for multiple sequence alignment (ClustalW [Thompson et al. 1994], PRANK [Löytynoja 2014], MAFFT [Katoh & Standley 2013], or MUSCLE [Edgar 2004]), (4) an application for homolog search (HMMER or the legacy version of BLAST), (5) CD‐Hit, and (6) Rate4Site.

## 4 CASE STUDY: CAV‐1

**FIGURE 2:**
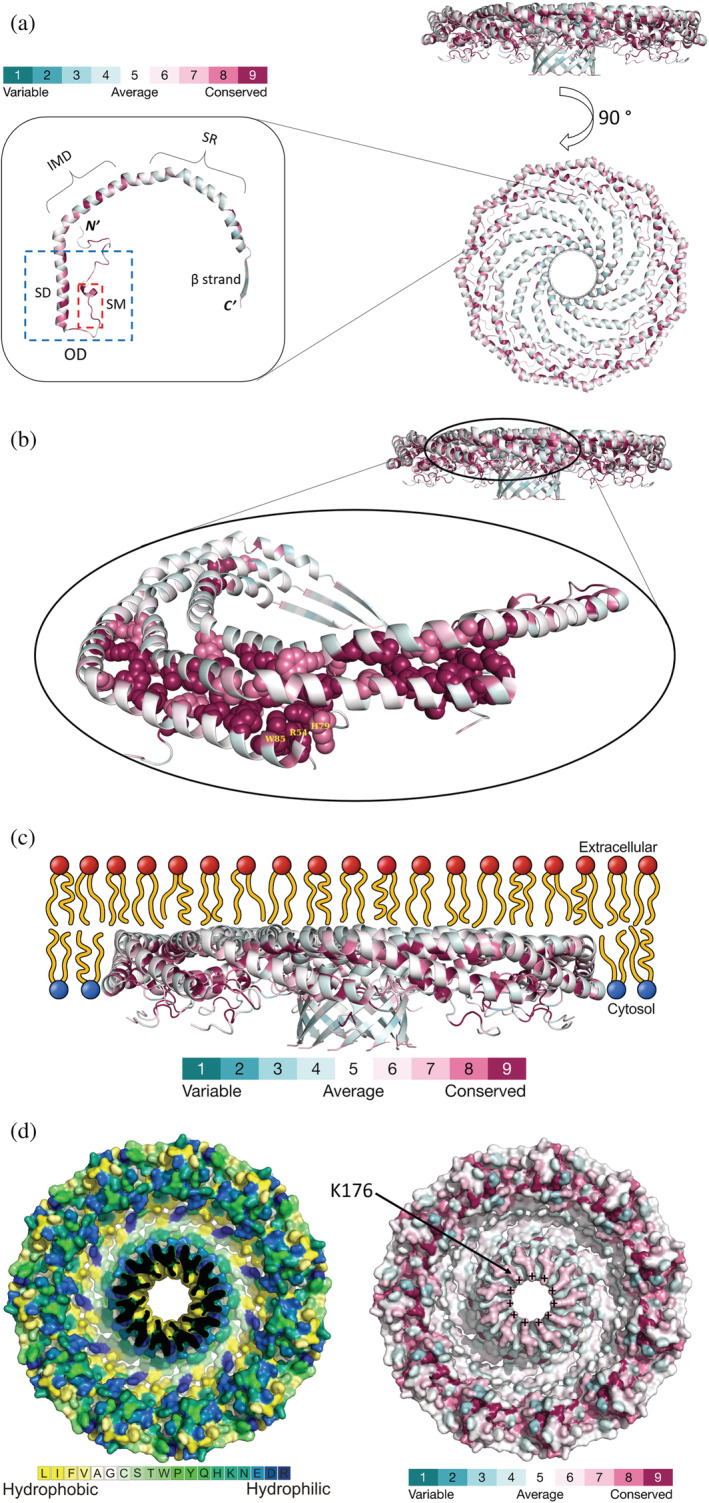
Structure, evolutionary conservation, and membrane binding of Cav‐1. The images show the cryo‐electron microscopy structure of Cav‐1 (PDB ID: 7sc0), solved at 3.40 Å resolution. (a) The overall structure and conservation of Cav‐1. Right: The entire 11‐mer complex, shown from two different angles. Left: A single monomer with the locations of the oligomerization domain (OD), scaffolding domain (SD), signature motif (SM), intramembrane domain (IMD), spoke region (SR), and the β strand marked. Cav‐1 is colored by ConSurf evolutionary conservation (see color scale), calculated with default settings (Ashkenazy et al. 2016) 300 homologues, and the empirical Bayesian algorithm (Mayrose et al. 2004). The homologues include all three caveolin isoforms: Cav‐1, which is expressed in most tissues, Cav‐2, which forms complexes with Cav‐1 but cannot independently form caveolae, and Cav‐3, which is expressed in muscles (Busija et al. 2017). (b) Evolutionarily conserved positions that mediate the interactions between Cav‐1 monomers. Top: The rim of the 8S structure, where most of the interfacial residues are located as part of the OD. Bottom: A blowup of this region, showing the interface between an arbitrarily selected monomer and its nearest neighbors from both sides. Interfacial residues with the highest ConSurf grades (8–9) are shown as spheres. R54, H79, and W85, which form interlocking interactions between two adjacent chains, are noted. (c) Association of Cav‐1 8S oligomers with the membrane as suggested by Ohi and co‐workers (Porta et al. 2022). The protein is shown as in panel b, top, and the lipid bilayer is shown schematically. (d) Left: Hydrophobicity of the β barrel's interior. A molecular surface view is shown from the cytoplasmic side, colored by the Kessel/Ben‐Tal hydrophobicity scale (Kessel & Ben‐Tal 2002; see color code at the bottom of the image). The edge of the barrel was removed to make the interior partially visible. Right: Surface representation of the evolutionary conservation pattern within the β barrel. The structure is shown as in the left image, except that it is colored by evolutionary conservation. The plus signs denote the positively charged amino groups of the conserved K176 in all 11 monomers.

An example of ConSurf's ability to capture functional characteristics of proteins is provided by analysis of caveolin‐1 (Cav‐1, Figure 2a, left). As the major protein of caveolae, which are functionally important membrane invaginations, Cav‐1 is crucial for various cellular processes such as endocytosis, membrane organization, lipid turnover, and the trafficking of cholesterol and proteins (Busija et al. 2017; Razani & Lisanti 2001; Rothberg et al. 1992). Cav‐1 is a monotopic membrane protein that traffics to the plasma membrane via the cell's secretory pathway. During this process, it inserts part way into the membrane of the endoplasmic reticulum, and oligomerizes into a large multi‐chain structure called 8S (Hayer et al. 2010; Kirkham et al. 2008; Porta et al. 2022). This oligomeric structure is then transported to the Golgi apparatus, and from there to the plasma membrane, where it binds other homo‐oligomers, as well as other proteins, to form a large network (Hayer et al. 2010; Schlegel & Lisanti 2000). The partial embedding of the Cav‐1 8S complexes inside the membrane induces the membrane to curve and form the caveolae invagination. The three‐dimensional structure of the 8S complex in detergent micelles has been determined recently by cryo‐electron microscopy (Porta et al. 2022; Figure 2a, right). The 8S complex is a mushroom‐like structure that consists of a large, flat alpha‐helical disk surrounding a central perpendicular beta‐barrel.

The per‐residue evolutionary conservation calculated by ConSurf revealed several important functional features of Cav‐1. First, the conservation pattern highlights the parts of Cav‐1 important for oligomer assembly and stabilization. Within the Cav‐1 8S structure, interactions between monomers are mediated primarily by the oligomerization domain, which includes (1) a scaffolding subdomain that is important for the binding of cholesterol (Yang et al. 2014) and proteins that are involved in transport and signaling (Mohan et al. 2015), and (2) a signature motif (Figure 2a, left). Most of the residues in the oligomerization domain that participate directly in inter‐chain interactions and are therefore responsible for the oligomerization are highly conserved (Figure 2b). These include, for example, R54 in the *N*‐terminal loop of one chain, which fits snugly into a pocket formed by H79 and W85 on an adjacent chain, thus “locking” the two chains together. Indeed, mutating R54 to alanine has been found to severely disrupt the formation of the 8S complex (Porta et al. 2022). The intramembrane domain, which mediates membrane binding and deformation (together with the scaffolding subdomain; Ariotti et al. 2015), also contains many conserved residues. The rest of the protein, which includes the spoke region and the β strand that forms the central barrel, are mostly variable. Interestingly, the last 10 residues of the protein have been implicated in oligomer‐oligomer binding (Schlegel & Lisanti 2000). Within this generally variable region, residues S168, V170, K176, and E177 are conserved (ConSurf grade of 7), suggesting that they may mediate interactions with other oligomers.

Another functional characteristic captured by ConSurf is regulation. For example, ConSurf assigns a conservation grade of 9 to S80, whose phosphorylation is crucial for Cav‐1 targeting to the endoplasmic reticulum (Schlegel et al. 2001). S168, which is also phosphorylated, has a slightly lower, but still above average, conservation grade of 7. This residue is included in the region of Cav‐1 that is involved in oligomer‐oligomer binding. Finally, the conservation pattern calculated by ConSurf also pinpoints certain residues that are mutated in various diseases. These include P132, which has a conservation grade of 9; replacement of P132 with leucine is associated with breast cancer and has been shown to promote metastasis (Bonuccelli et al. 2009). This essentially invariant proline is located between the intramembrane domain and spoke region, forming a kink that separates the domains. Furthermore, prolines within alpha‐helices of membrane proteins have been proposed to act as hinges that facilitate conformational changes (Sansom & Weinstein 2000). It is possible that P132 fulfills a similar role, perhaps as part of Cav‐1's ability to deform and curve the membrane. Replacement of P132 with leucine is expected to interfere with these functions. It is worth noting that P123, despite being far from the oligomerization domain, forms tight contacts with residues in two adjacent chains. Introducing the large side chain of leucine might lead to clashes with adgacent residues, thus disrupting oligomer stability and organization. In any case, the invariance of P123 stands out within a non‐conserved region in Cav‐1 as an indication of its importance.

It is important to note that the use of ConSurf to detect functional features is case‐dependent as not all biological features are necessarily shared by all the homologues. For example, cysteines 133, 143, and 156, which are palmitoylated in caveolins (Dietzen et al. 1995), and are therefore functionally important, are assigned an average conservation grade of 5. It may be that certain caveolin types do not undergo palmitoylation or they may undergo this modification on different positions. It is noteworthy that the palmitoylation does not seem to be important for the localization of Cav‐1 to caveolae (Dietzen et al. 1995), although it might reduce its membrane affinity (Krishna & Sengupta 2019; Monier et al. 1996) and affect signaling in certain tissues (Lee et al. 2001; Schianchi et al. 2020). Other examples of functional residues that are assigned low conservation grades by ConSurf include those that undergo ubiquitination and SUMOylation (Bakhshi et al. 2013; Kirchner et al. 2013). Ubiquitination occurs only in Cav‐1, whereas SUMOylation occurs only in Cav‐3. Thus, it is important to know the biology of the query protein (and homologues) when using its conservation pattern to detect functionally important residues and regions.

The determinants of Cav‐1 orientation in the membrane and the principles that underly its induction of membrane curvature are not entirely clear yet. Based on the position of the detergent micelle in the density map and their previous negative stain analysis (Han et al. 2020), Ohi et al. concluded that the 8S complex partitions into the cytoplasmic leaflet of the membrane with the flat disk embedded in the membrane and the β‐barrel facing the cytoplasm (Figure 2c; Porta et al. 2022). They suggested that partitioning into the cytoplasmic leaflet of the membrane allows Cav‐1 to induce the changes required for forming caveolae. The central beta‐barrel forms a hydrophobic pore that leads from the hydrocarbon core of the membrane to the cytoplasm (Figure 2d, left). The pore has a diameter of 15 Å at its narrowest point (measured between side chain ends), and Ohi and co‐workers suggested that it may shuttle individual lipid molecules between the membrane and the cytoplasm (Porta et al. 2022). Interestingly, the cytoplasmic edge of the beta‐barrel is partially capped by K176 (Figure 2d, right), creating a charge density that separates the pore interior from the cytoplasm. We speculate that this conserved residue (assigned a conservation grade of 7), which resides within a random coil and has a long flexible side chain, functions as a gate. That is, when the K176 side chain faces the pore center, it may prevent passage of lipid molecules between the barrel and cytoplasm. A local conformational change may open this gate (i.e., point the side chain of K176 away from the center of the barrel), allowing lipids to go through.

## 5 DISCUSSION

Here, we have summarized the main improvements in ConSurf since the last report in 2016 (Ashkenazy et al. 2016) and demonstrated its use in pinpointing key functional regions in a recently determined structure of the 8S homo‐oligomeric structure of Cav‐1. Validating the utility of conservation analysis, some of the highly conserved amino acid positions of Cav‐1 are known to be important for function. The high conservation of known functional residues, whose importance was revealed in experiments or simulations in a specific protein (or nucleic acid), indicates that the function is shared among other members of the family.

Most interesting, however, are highly conserved positions whose functional roles are yet to be discovered. In this respect, ConSurf analysis is a powerful evolutionary flashlight that can be used to guide experiments and simulations of proteins or nucleic acids. Using a structure as query is much preferred over a sequence‐based query because the structural context makes it easier to develop testable hypotheses.

Sequence diversity is very important in evolutionary analysis. Thus, conducting a ConSurf calculation with too few homologues might not be particularly informative. This is also relevant when starting a second ConSurf analysis with a selected sub‐tree. Even though the pipeline allows analysis of as few as five homologues (including the query), ConSurf calculations with <50 homologues is not recommended. In principle, prediction accuracy increases as more homologues are included. However, to keep computational burden modest, we recommend that no more than 300 homologues be used.

### 5.1 Comparison of the old and new pipelines

The conservation grades calculated by the new pipeline are not identical to those reported by the previous version of the pipeline. For the most part, the differences can be traced to CD‐HIT. The heuristic approach used in CD‐HIT for clustering the homologues and choosing representatives is sensitive to the order of the input data. That is, when the “unique homologues” are sorted differently, CD‐HIT results differ. This, in turn, leads to differences in evolutionary rates calculated by Rate4Site. The new pipeline sorts the homologues by the *E*‐values of their similarity to the query, whereas the previous pipeline stored them in a hash table as a disordered list of objects. Encouragingly, our comparison showed that, for the most part, the differences are not significant, considering the confidence assigned to each of the conservation grades.

### 5.2 Limitations and outlook

When starting with a query protein sequence, ConSurf may use HHpred or MODELLER to produce a homology model. However, it makes sense to present the conservation patterns on AlphaFold model structures. Currently, the pipeline automatically searches the AlphaFold database for a model structure when starting either from the UniProt ID or from a protein sequence. We have created the ConSurf‐DB repository that includes pre‐calculated evolutionary profiles of most proteins in the PDB (Ben Chorin et al. 2020). We update this database frequently to include new structures, and we aim to include AlphaFold models in the near future. This will make available pre‐computed conservation profiles for virtually all UniProt proteins. This aim is obviously very demanding because there are hundreds of millions of proteins. We will thus prioritize this task according to taxonomic classification (e.g., all human proteins) or other research‐oriented ordering. In addition, we are in the process of accelerating the C++ encoding of Rate4Site to make it computationally more efficient. Currently, the standalone version of ConSurf can only read PDB files and cannot parse the heavier mmCIF format. MMseqs2 has not yet been implemented in the standalone version of ConSurf. Both the web server and standalone versions of ConSurf are incompatible with the newest builds of BLAST+ and only work with legacy versions of NCBI BLAST.

## AUTHOR CONTRIBUTIONS

**Barak Yariv:** Methodology (lead); software (lead). **Elon Yariv:** Methodology (supporting); software (supporting); writing – original draft (supporting). **Amit Kessel:** Formal analysis (equal); investigation (lead); writing – original draft (supporting); writing – review and editing (supporting). **Gal Masrati:** Conceptualization (equal); methodology (supporting); software (supporting); validation (supporting); visualization (lead); writing – original draft (supporting); writing – review and editing (supporting). **Adi Ben Chorin:** Conceptualization (equal); methodology (supporting). **Eric Martz:** Methodology (supporting); software (supporting); validation (supporting); visualization (lead); writing – original draft (supporting); writing – review and editing (supporting). **Itay Mayrose:** Conceptualization (supporting); methodology (supporting); resources (supporting); software (supporting); writing – original draft (supporting); writing – review and editing (supporting). **Tal Pupko:** Conceptualization (supporting); methodology (supporting); software (supporting); writing – original draft (supporting); writing – review and editing (supporting). **Nir Ben‐Tal:** Conceptualization (lead); methodology (supporting); software (supporting); visualization (supporting); writing – original draft (lead); writing – review and editing (lead).

## Data Availability

The ConSurf web‐site is available at: https://consurf.tau.ac.il; The standalone pipelines is available at: https://consurf.tau.ac.il/STANDALONE/stand_alone_consurf‐1.00.rar
